# Clinical presentation of neurosyphilis – a single-center retrospective data analysis

**DOI:** 10.1186/s42466-026-00497-1

**Published:** 2026-04-30

**Authors:** Susanne Dyckhoff-Shen, Ilias Masouris, Stefanie Völk, Konstantin Pusl, Ulrich Seybold, Matthias Klein

**Affiliations:** 1 Department of Neurology, LMU University Hospital, LMU Munich, Marchioninistr. 15, Munich, D-81377 Germany; 2 Division of Infectious Diseases, Department of Medicine IV and Center for Clinical Infectious Diseases, LMU University Hospital, LMU Munich, Munich, Germany

**Keywords:** Neurosyphilis, HIV, Treponema pallidum

## Abstract

**Background:**

As cases of neurosyphilis are rising worldwide – especially in people living with HIV (PLWH) – current data on clinical presentation, diagnostics and management of neurosyphilis in Europe are of high interest.

**Methods:**

Clinical data from adult patients, who had been treated for neurosyphilis at a university hospital in Germany from 2005 to 2024, were retrospectively analyzed. Probable diagnosis was based on corresponding signs and symptoms, positive syphilis serology, Cerebrospinal fluid (CSF) abnormalities, and/or improvement after antibiotic therapy. In asymptomatic patients, diagnosis was based on CSF abnormalities with positive serology. A positive CSF/serum treponemal antibody index confirmed the diagnosis.

**Results:**

77 patients with neurosyphilis were identified, the majority being male (95%), with a high proportion of HIV co-infection (43%). Clinical presentation was most frequently asymptomatic or ocular neurosyphilis (both 26%), other common symptoms were cognitive deficits, headache, and psychiatric symptoms. CSF abnormalities and additional co-infections were more pronounced among PLWH. CSF cell count and protein concentration were significantly lower in asymptomatic (latent) neurosyphilis compared to symptomatic cases, yet between various symptomatic manifestations of neurosyphilis only minor differences were observed. CSF VDRL-test was negative in nearly half of patients with confirmed neurosyphilis. Penicillin G was the predominant treatment (71%), with ceftriaxone used as the main alternative (21%), and doxycycline in 3 patients mostly due to penicillin allergy. Clinical improvement was observed in 88% of symptomatic patients.

**Discussion:**

Our findings align with recent reports describing neurosyphilis as a heterogeneous infection strongly linked to HIV-infection. The limited sensitivity of CSF VDRL reinforces the need for multimodal diagnostics. Current CSF testing criteria may miss asymptomatic cases, warranting heightened clinical vigilance.

**Supplementary Information:**

The online version contains supplementary material available at 10.1186/s42466-026-00497-1.

## Introduction

 Neurosyphilis is the neurological manifestation of syphilis, an infection with the mostly sexually transmitted bacterium *Treponema pallidum*. This condition can affect various parts of the central nervous system (CNS). A distinction is made between “early neurosyphilis”, which develops months to a few years after the primary infection, and “late neurosyphilis”, which can occur many years or even decades later [1]. Although neurosyphilis was once considered a rare complication in the antibiotic era [2], its incidence appears to be increasing again in many countries, paralleling the resurgence of syphilis [3–6]. Epidemiological data from the Robert Koch Institute (RKI) indicate that syphilis infection rates have been increasing in Germany significantly since 2010 and were already rising slowly before that [1, 7], particularly among high-risk populations such as men who have sex with men (MSM) and people living with HIV (PLWH), in whom neurosyphilis is frequently underdiagnosed. In cases of immunosuppressed status, these patients are at increased risk of developing neurosyphilitic complications [8]. A low CD4 + T-cell count and high HIV viral load are considered factors that further elevate the risk of neurological involvement.

Despite the availability of effective antibiotic treatments, neurosyphilis still presents significant clinical challenges, particularly in high-risk groups such PLWH. The disease often presents with subtle or non-specific symptoms, which may lead to delayed diagnosis and, consequently, increased morbidity [3]. As “the great mimicker” [3], the clinical presentation of neurosyphilis is highly variable, ranging from cognitive impairment (e.g., dementia, confusion) and meningovascular manifestations resembling stroke, to motor deficits such as paresis and coordination disorders (as seen in tabes dorsalis). Psychiatric symptoms, including depression and psychosis, may also occur. In some cases, asymptomatic meningitis with inflammatory CSF findings but without overt neurological symptoms may be present [4].

The broad spectrum of symptoms makes it a challenge to correctly diagnose neurosyphilis patients, especially as the diagnosis relies on a summary of clinical presentation, serum syphilis tests, and CSF parameters without one single reliable parameter. Several scores with diagnostic criteria exist, but an internationally well recognized diagnostic score is lacking [9].

Our retrospective study aimed to conduct a comprehensive analysis of clinical, diagnostic, and therapeutic characteristics of adult patients diagnosed with neurosyphilis at the University Hospital of Munich between January 1, 2005, and December 31, 2024. This investigation seeked to provide valuable insights on diagnostic challenges and therapeutic outcomes of neurosyphilis, thereby contributing to improved patient care and evidence-based clinical practice.

## Study population and methods

**Fig. 1:**
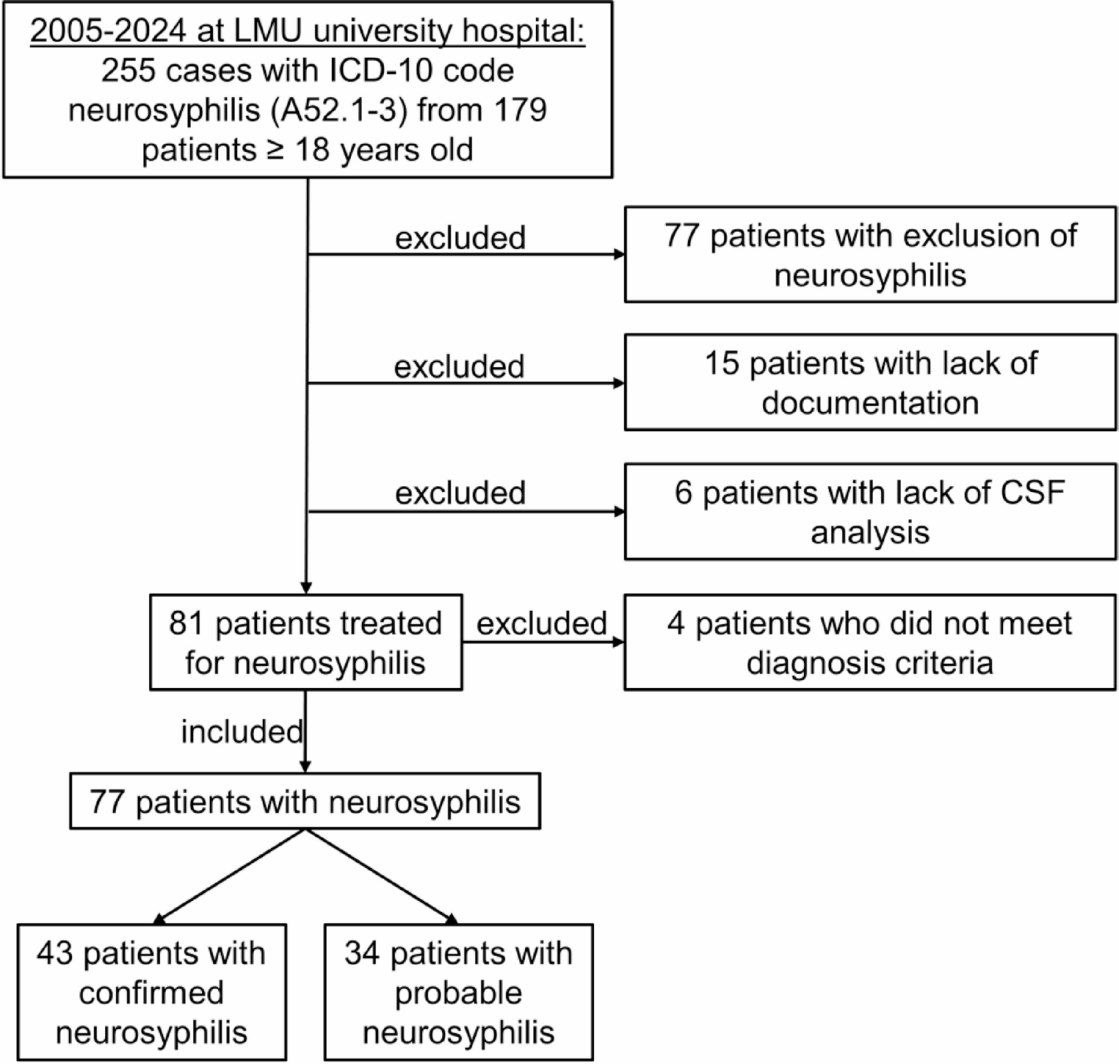
Inclusion diagram

This study was designed as a single-center, retrospective cohort study. It was conducted at the LMU University Hospital of Munich, a tertiary care academic institution. The study period spanned from January 1, 2005, to December 31, 2024. All patients aged ≥ 18 years who were diagnosed with neurosyphilis during inpatient care within the study period were eligible for inclusion. The screening diagnosis was based on the corresponding DRG coding (ICD-10 A52.1-3) for the respective patient cases and was double-checked by clinical experts for correct diagnosis. In the study period, 255 cases of neurosyphilis among 179 patients were documented. After manual check of correct diagnosis in patients reports, 77 patients in whom neurosyphilis was ruled out, were excluded. Moreover, 15 patients with incomplete documentation and 6 patients with lack of CSF analysis were excluded. In total, 81 patients were identified who had been treated for neurosyphilis. As a next step, those neurosyphilis cases were classified as probable or confirmed according to the established criteria from the German Neurology Society (DGN) guideline for neurosyphilis [10]: (1) subacute or chronic clinical neurological or psychiatric signs or symptoms consistent with neurosyphilis, (2) CSF abnormalities, e.g. pleocytosis, elevated total protein or blood-CSF barrier dysfunction, (3) favorable impact of antibiotic treatment on clinical symptoms and/or cerebrospinal fluid (CSF) abnormalities and (4) reactive syphilis serology in serum. For probable neurosyphilis, 2 criteria from (1) to (3) and criterium (4) had to be fulfilled. Evidence of intrathecal *Treponema pallidum* antibody production led to confirmed neurosyphilis diagnosis. Four cases did not fulfill the diagnostic criteria (asymptomatic cases with normal CSF parameters) and were therefore excluded from the study. In total, 77 patients were included with 34 cases classified as probable (30 with negative CSF/serum antibody index and 4 without determined antibody index) (Fig. 1) and 43 cases classified as confirmed [10].

Data were extracted from electronic medical records. The following parameters were obtained: demographics (age, gender, sexual orientation), clinical presentation (neurological and psychiatric symptoms), comorbidities including HIV status, diagnostic findings (serum and CSF syphilis serology, CSF cell count, total protein, glucose, albumin quotient, oligoclonal bands (OCB), and MRI findings), therapy (antibiotic regimen), complications, and outcome.

### Statistical analysis

Statistical analysis was performed using GraphPad Prism and SPSS Statistics 29. The principal statistical tests were one way analysis of variance (ANOVA) and subsequent Bonferroni post-hoc tests, student’s t-test/Mann-Whitney test and Chi^2^ test. Differences were considered significant at *p* < 0.05.

## Results

### Predominantly male cohort with high proportion of HIV co-infection

**Fig. 2:**
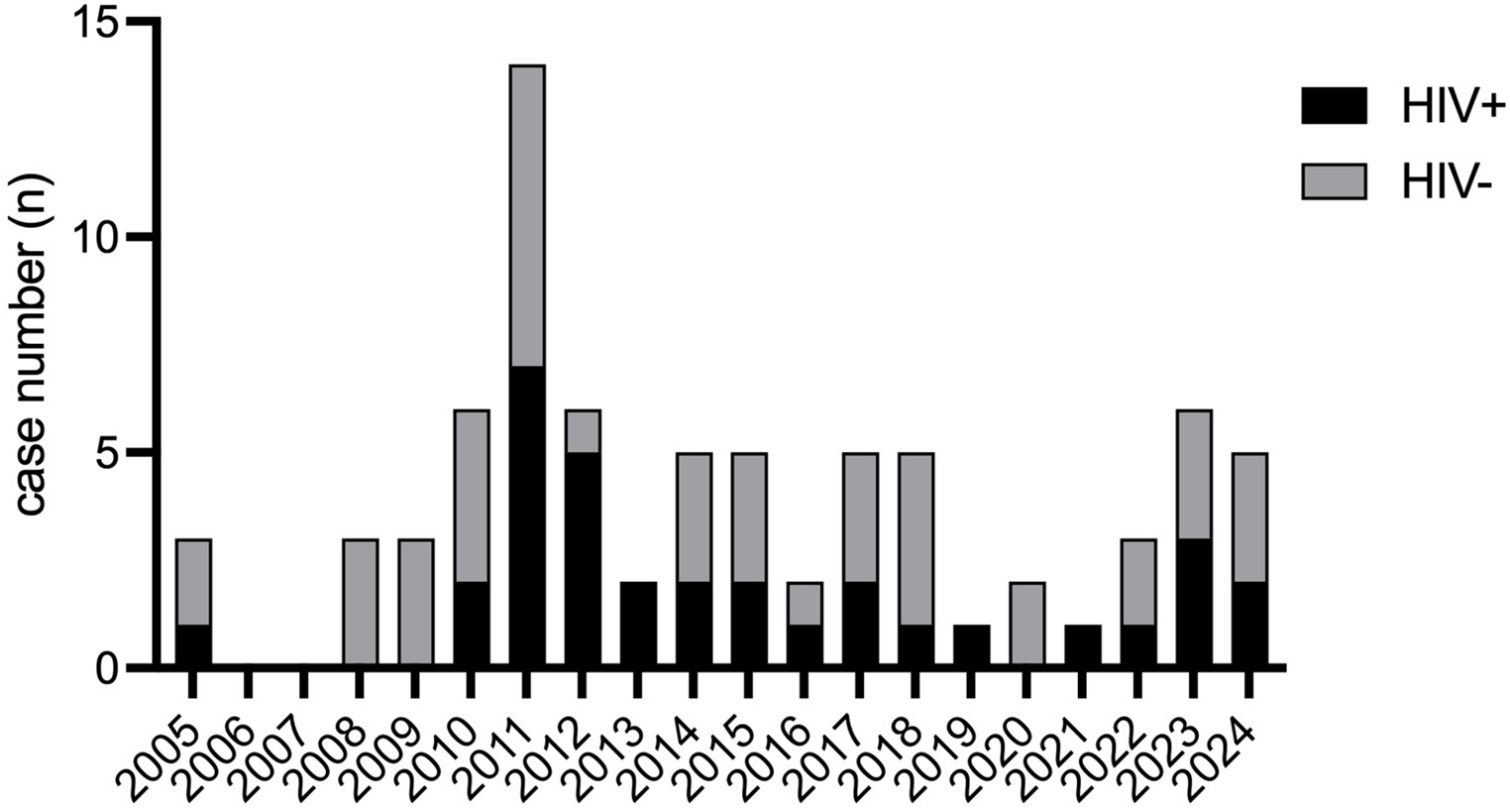
Case distribution over study period. Cases were categorized according to HIV-status

We identified 77 patients diagnosed with neurosyphilis and treated at our institution between 2005 and 2024. The temporal distribution was heterogenous, with a peak in 2011 and no cases documented in 2006 or 2007 (Fig. 2). The majority of patients were (cis-)male (95%), with only four (cis-)women and – as far as can be ascertained from the partially limited documentation, there was no trans-person included among the patients. The median age at diagnosis was 47 years. Thirty-three individuals (43%) were living with HIV, and more than one quarter (29%) had previously been diagnosed and treated for syphilis. Among the female patients, none were HIV-positive. Documentation of sexual preference was only available in 29 men, of whom 27 were MSM and 2 men who have sex with women (MSW).

### Asymptomatic or ocular neurosyphilis as most common clinical presentation

Clinical presentation was heterogenous (Table 1). The most common manifestations were visual impairment (26%), cognitive deficits (25%), headache (21%) and psychiatric symptoms (20%). Less frequent symptoms included gait ataxia, sensory loss, limb weakness, cranial nerve palsies, and seizures. One quarter of patients were (neurologically) asymptomatic (26%). Several patients also presented with concomitant cutaneous or genital syphilitic lesions. Comparison of symptom frequency revealed slightly higher frequency of headache, cognitive deficits, limb weakness, gait ataxia, seizures, and psychiatric symptoms among HIV-positive compared to HIV-negative patients – although without significant difference, except for the presence of seizures ad admission (3 vs. 0 cases).

**Table 1:** Patient characteristics and clinical presentation in total cohort and depending on HIV status

Baseline characteristics	HIV-positive (*n* = 33)	HIV-negative (*n* = 44)	All patients (*n* = 77)
Age, years, median (IQR)	49 (42–57)	46 (41–55)	47 (41–55)
Sex, male, n (%)	33 (90.9%)	40 (100%)	73 (94.8%)
Previous syphilis, n (%)	8 (24.2%)	14 (31.8%)	22 (28.6%)
MSM, n (%)	16/16 (100.0%)	11/13 (84.6%)	27/29 (93.1%)^1^
**Symptoms**,** n (%)**			
Asymptomatic	6 (18.2%)	14 (31.8%)	20 (26.0%)
Headache	9 (27.3%)	7 (15.9%)	16 (20.8%)
Cognitive deficit	10 (30.3%)	9 (20.5%)	19 (24.7%)
Cranial nerve palsy	2 (6.1%)	3 (6.8%)	5 (6.5%)
Limb weakness	5 (15.2%)	2 (4.5%)	7 (9.1%)
Sensory loss	7 (21.2%)	3 (6.8%)	10 (13.0%)
Gait ataxia	7 (21.2%)	4 (9.1%)	11 (14.3%)
Seizures	3 (9.1%)* ^**2**^	0 (0%)* ^2^	3 (3.9%)
Visual impairment	5 (15.2%)	15 (34.0%)	20 (26.0%)
Psychiatric symptoms	9 (27.3%)	6 (13.6%)	15 (19.5%)
Abnormal neurological examination	18 (50.0%)	15 (33.3%)	33 (40.7%)
Skin lesions	3 (9.1%)	6 (13.6%)	9 (11.7%)
Genital lesions	3 (9.1%)	5 (11.4%)	8 (10.4%)
**Neurosyphilis manifestation**			
Early meningitis	4 (12.1%)	3 (6.8%)	7 (9.1%)
Meningovascular neurosyphilis	9 (27.3%) * ^**3**^	3 (6.8%) * ^**3**^	12 (15.6%)
Ocular neurosyphilis	5 (15.2%) * ^**3**^	15 (34.1%) * ^**3**^	20 (26.0%)
Tabes dorsalis	4 (12.1%)	2 (4.5%)	6 (7.8%)
General paresis	5 (15.2%)	7 (15.9%)	12 (15.6%)
Asymptomatic neurosyphilis	6 (18.2%)	14 (31.8%)	20 (26.0%)

MSM: men who have sex with men. ^1^Documentation on sexual orientation was available for 29 men. Statistical tests were student’s t-test, Mann-Whitney test, or Chi-square test, as applicable, * *p* < 0.05 (^2^ *p* = 0.041, ^3^ *p* < 0.05)

Symptom duration before admission varied widely, ranging from as little as one day – in one case of a stroke and one case of papillitis due to neurosyphilis – to more than five years in patients with ataxia and vertigo. Based on patient history and clinical presentation, cases were classified into six categories of neurosyphilis: (1) early meningitis (*n* = 7), (2) meningovascular neurosyphilis (*n* = 12), (3) ocular neurosyphilis (*n* = 20), (4) tabes dorsalis (*n* = 6), (5) general paresis (*n* = 12), and (6) asymptomatic (*n* = 20). Among the four female patients in the cohort, one presented with early meningitis, two with ocular neurosyphilis, and one with tabes dorsalis. HIV-negative patients presented more frequently with ocular neurosyphilis than HIV-positive patients whereas HIV-positive patients had significantly higher cases of meningovascular neurosyphilis.

In most cases (46/77), patients were admitted with suspected neurosyphilis following a current or prior diagnosis of cutaneous or genital syphilis. In the remaining cases, the most common differential diagnoses at admission were chorioretinitis/uveitis/papillitis (*n* = 11), optic neuritis (*n* = 5), dementia (*n* = 3), meningitis (*n* = 2), depression (*n* = 2) and polyneuropathy (*n* = 2), ALS (*n* = 1), spinal disc compression (*n* = 1), neuroborreliosis (*n* = 1), ataxia (*n* = 1), stroke (*n* = 1), vestibular schwannoma (*n* = 1).

### CSF abnormalities and co-infections more pronounced in HIV-positive patients

CSF analysis revealed a mean pleocytosis of 38 cells/µl with 78% lymphocytes, accompanied by elevated protein concentration and normal glucose index (Table 2). Serum parameters showed a normal median C-reactive protein (CRP) of 0.45 mg/dl (reference < 0.5 mg/dl) and a normal median leukocyte count (6.6 G/l).

Treponemal serological testing demonstrated universal reactivity: Serum *Treponema pallidum* particle agglutination (TPPA) was positive in all patients, the fluorescent treponemal antibody absorption test (FTA-ABS) was positive in all 55 tested patients, and the rapid plasma regain card test (RPR) test was positive in 87%. In the CSF, syphilis testing using TPPA was positive in all 56 tested patients, and FTA-ABS in 40 of 41. CSF Venereal Disease Research Laboratory (VDRL) testing was performed in 32 patients and was reactive in 13 (41%). The neurosyphilis antibody index (CSF/serum) was available for 73 patients, with a median value of 3.0.

Comparison of basic CSF parameters between HIV-positive and HIV-negative patients showed significant differences: the HIV-positive subgroup had higher cell counts, total protein concentrations, albumin index values, and a greater frequency of positive oligoclonal bands (OCB), whereas CSF glucose and glucose index were significantly lower. Serum parameters and syphilis serologic test results in both CSF and serum did not differ significantly between the two groups.

**Table 2:** Diagnostic findings including CSF analysis, serum parameters, syphilis serology, other pathogens and cMRT findings

Diagnostic findings	HIV-positive (*n* = 33)	HIV-negative (*n* = 44)	All patients (*n* = 77)
**CSF parameters**			
Cells/µl, median (IQR)	10 (5–88)*	9 (5–19)*	9 (5–43)
Lymphocytes (%), median (IQR)	**88.5 (81.3–93** ***)*** *	74 (64.5–85)*	83 (67–89)
Total protein (mg/dl), median (IQR)	70.1 (45.7–90.8)*	48.4 (36.5–65.5)*	55.0 (38.7–71.8)
Albumin index, median (IQR)	10.3 (7.9–14.8)**	7.3 (4.9–9.8)**	8.0 (5.3–11.1)
OCB^1^ pos., n (%)	20/23 (87.0%) *	25/41 (61.0%) *	45/64 (70.3%)
Glucose (mg/dl), median (IQR)	56.1 (49.3–62)**	62.9 (60.0-69.6)**	61.6 (55.8–67.6)
Glucose-index, median (IQR)	0.49 (0.40–0.57)***	0.61 (0.55–0.70)***	0.57 (0.47–0.67)
TPPA pos., n (%)	29/29 (100%)	27/27 (100%)	56/56 (100%)
FTA-Abs pos., n (%)	21/21 (100%)	19/20 (95.0%)	40/41 (97.6%)
VDRL pos., n (%)	7/17 (41.2%)	6/15 (40.0%)	13/32 (40.6%)
Lues IgG index, median (IQR)	3.6 (1-13.1)	2 (1-8.7)	3 (1-10.9)
**Serum parameters**			
CRP (mg/dl), median (IQR)	0.38 (0.2–1.8)	0.46 (0.2–2.1)	0.45 (0.2–1.9)
Leukocytes (G/l), median (IQR)	6.1 (4.9–7.2)	7 (5.4–9.4)	6.6 (5.0-8.3)
TPPA pos., n (%)	33/33 (100.0%)	44/44 (100.0%)	77/77 (100.0%)
FTA-Abs pos., n (%)	27/27 (100.0%)	28/28 (100.0%)	55/55 (100.0%)
Cardiolipin-RPR pos., n (%)	28/33 (84.8%)	36/41 (87.8%)	64/74 (86.5%)
**Additional pathogens**	6 (18.2%) *	1 (2.3%) *	7 (9.1%)
**cMRI abnormalities**, n (%)	9/15 (60.0%)	5/18 (27.8%)	14/33 (42.4%)
**Diagnostic criteria fulfilled**			
Probable neurosyphilis	12 (36.4%)	22 (50.0%)	34 (44.2%)
Confirmed neurosyphilis	21 (63.6%)	22 (50.0%)	43 (55.8%)

Statistical tests were student’s t-test, Mann-Whitney test or Chi-square test as applicable, * *p* < 0.05, ** *p* < 0.01, *** *p* < 0.001. ^1^oligoclonal bands. IQR= interquartile range

Coinfections with pathogens other than *Treponema pallidum* and HIV were identified in seven patients. HIV-positive patients had a significantly higher risk of concurrent infection than HIV-negative patients (6 vs. 1 case). The additionally detected pathogens included *Toxoplasma gondii* (*n* = 3, positive serology), *Cryptococcus neoformans* (*n* = 1, positive PCR, culture and microscopy in CSF), *Pseudomonas aeruginosa* (*n* = 1, positive blood culture), *N. gonorrhoeae*,* Mycoplasma hominis*, and *Chlamydia trachomatis* (*n* = 1, positive PCR in urine and rectal swab) and *Mycoplasma pneumoniae* and *Campylobacter jejuni* (*n* = 1, positive serology). In particular, additional CNS infection with toxoplasmosis or cryptococcal meningitis may be confounders of clinical data, CSF parameters, or therapeutical outcome in this study cohort. Those four coinfections all occurred in patients with newly diagnosed or untreated HIV.

Magnetic resonance imaging (MRI) was conducted in 33 patients. Of these, 14 (42%) showed radiological abnormalities. In HIV-positive patients (*n* = 9), imaging findings such as meningeal enhancement, contrast-enhancing lesions, acute ischemic lesions, and multifocal signal alterations were most often attributed to HIV-related comorbidities, including toxoplasmosis or progressive multifocal leukoencephalopathy (PML). In HIV-negative patients, MRI abnormalities consisted of cortical atrophy (*n* = 2; aged 63 and 79 years), older ischemic lesions (*n* = 2; aged 54 and 67 years) and there was one case of a 30-year-old patient with a contrast lesion of the right vestibulocochlear nerve due to neurosyphilis, who presented with hearing loss, ear pain, and a peripheral vestibular deficit on the right side.

### Milder CSF abnormalities in asymptomatic neurosyphilis

Generally, patients with late neurosyphilis were older than those in early stages. Specifically, patients with general paresis were significantly older than those with meningovascular, ocular, or asymptomatic neurosyphilis. When comparing CSF parameters between neurosyphilis manifestations, asymptomatic patients had significantly lower CSF white cell count, protein concentration, and albumin quotients compared to the symptomatic cohort. Moreover, CSF cell count tended to be higher in early meningitis, meningovascular, ocular neurosyphilis, and general paresis than in tabetic patients (Supplemental Table 1). Lues antibody index tended to be more pronouncedly increased in meningovascular and ocular neurosyphilis as well as general paresis than in the other manifestations. Patients with general paresis had significantly more often MRI abnormalities than those with ocular neurosyphilis.

The subgroup of 20 (neurologically) asymptomatic patients were all male with a mean age of 46.6 years younger than symptomatic patients. All were admitted with suspected neurosyphilis due to recent diagnosis of syphilis with 12 showing a penile ulcus and/or syphilitic exanthema. Seven had previously been diagnosed with a syphilis infection and six were HIV-positive (2x viral load below detection limit, 1x above, 3x not determined). Half of these patients had either a reinfection or continuously elevated serum titres despite antibiotic therapy which might have led to more extensive diagnostic including CSF analysis. MRI was conducted in two asymptomatic patients without any abnormalities. CSF analysis revealed mild lymphocytic pleocytosis with a median cell count of 9/µl, and median protein concentration of 44 mg/dl. All but two asymptomatic neurosyphilis patients were treated with penicillin G i.v. for 14 days, one with doxycycline, one with piperacillin/tazobactam, and none with ceftriaxone.

### Clinical improvement after antibiotic therapy despite lack of intrathecal treponemal antibody synthesis

Overall, neurosyphilis diagnosis was categorized as confirmed in 43 cases and probable in 34. The subgroup classified as probable neurosyphilis did not demonstrate an elevated CSF/serum treponemal antibody index. In four of 34, the index was not determined, while in the remaining 30 patients, the index was negative (< 1.5). The diagnosis of neurosyphilis in patients with a negative antibody index is inherently uncertain. We therefore examined these 30 cases in greater detail. Eight patients were asymptomatic but exhibited positive syphilis serology together with abnormal CSF parameters. TPPA and/or FTA-ABS in CSF were positive in five of these patients and not determined in three. In one 26-year-old, neurologically asymptomatic patient with a penile ulcus that had been treated 17 months prior with doxycycline, neither CSF parameters nor serum RPR improved after i.v. penicillin G, suggesting persistent residual titers rather than active disease. The remaining 22 symptomatic patients with negative antibody index all had CSF pleocytosis and/or elevated protein concentrations. Significant clinical improvement was seen after treatment in 15/22 patients.

In 15 of the 22 symptomatic patients (68%), symptom duration ranged from 1 day to 2 months at the time of diagnosis, raising the possibility that intrathecal antibody production had not yet developed at the time of lumbar puncture in those cases.

### CSF VDRL negative in almost 50% of cases with confirmed neurosyphilis

The subgroup of 43 confirmed cases included 2 female patients and half were HIV-positive (*n* = 21). Ten patients were asymptomatic, the remaining 33 were symptomatic: early meningitis (*n* = 2), meningovascular neurosyphilis (*n* = 8), ocular (*n* = 11), tabes dorsalis (*n* = 3) and general paresis (*n* = 9). Median CSF cell count was 22 cells/µl and median CSF protein concentration was 62 mg/dl. The median CSF/serum antibody index was 8.0 (IQR 4.0-19.6). In 22 patients classified as confirmed neurosyphilis, VDRL in CSF was performed yielding a negative result in almost half (10/22). MRI was abnormal in 11/23 patients with confirmed neurosyphilis. Three patients had additional CNS coinfections (1x cryptococcal meningitis, 2x CNS toxoplasmosis).

### Normal CSF parameters more common in late neurosyphilis

A total of 17 patients in the total cohort revealed normal CSF cell count (< 5 cells/µl), including five who also exhibited normal CSF protein concentrations. All five patients – except for one - who did not meet the standard CSF criteria nonetheless showed positive treponemal serology (TPPA and FTA-ABS) as well as an elevated CSF/serum antibody index. Four of these five patients were HIV-positive. They presented with typical neurological and or psychiatric symptoms – categorized as general paresis (*n* = 4) or meningovascular neurosyphilis (*n* = 1). Those patients were all male, aged 50–67 years, and reported symptom durations ranging from several weeks to several years. Four demonstrated positive CSF TPPA, FTA-ABS and elevated CSF/serum antibody index; two of them showed clinical improvement upon intravenous antibiotic treatment. The single HIV-negative patient, who had been diagnosed and treated for syphilis 10 years prior, displayed negative CSF OCB and a negative antibody index, suggesting residual serological activity rather than active neurosyphilis; however, he experienced clinical improvement after treatment with intravenous penicillin G.

### Penicillin G remains predominant treatment, ceftriaxone main alternative

Most patients were treated with penicillin G (*n* = 55, 71%), followed by ceftriaxone which was used in 21% of cases (Table 3). In total, three patients received doxycycline as antibiotic therapy for neurosyphilis: in two cases, a penicillin allergy was documented explaining the use of doxycycline; for the third patient, the treatment decision for doxycycline was not evident from the patient charts. Two patients received piperacillin/tazobactam and for another one, who left the clinic against medical advice, the antibiotic agent was not documented.

Analyzing the relation between administered antibiotic and clinical improvement, we initially observed a higher proportion of improvement in the ceftriaxone group. Interestingly, asymptomatic patients were never treated with ceftriaxone leading to this result. When we excluded asymptomatic patients, this difference in clinical improvement disappeared. Five patients with long lasting symptoms such as dementia (*n* = 2), chronic vertigo and sensory deficits (*n* = 1), chronic chorioretinitis (*n* = 1), and chronic headache (*n* = 1) did not experience clinical improvement after antibiotic therapy.

**Table 3:** Antibiotic therapy was predominantly penicillin G, followed by ceftriaxone

Treatment	Penicillin G (*n* = 55)	Ceftriaxone (*n* = 16)	Doxycycline (*n* = 3)
Age, years, median (IQR)	47.0 (41–55)	46.0 (40–59)	45.0 (37–45)
Sex, male, n (%)	53 (96.4%)	14 (87.5%)	3 (100.0%)
HIV-positive	24 (43.6%)	7 (43.8%)	1 (33.3%)
**Neurosyphilis manifestation**			
Early meningitis	5	2	0
Meningovascular type	9	2	0
Ocular neurosyphilis	11	8	0
Tabes dorsalis	3	2	1
General paralysis	9	2	1
Asymptomatic neurosyphilis	18	0	1
Clinical improvement	24/29 (82.8%)	15/15 (100.0%)	2/2 (100.0%)
CSF improvement	14	6	1
Serum improvement	17	3	0

Treatment groups were analyzed based on their demographic characteristics, neurosyphilis manifestation, and outcome. Three other patients were treated with piperacillin/tazobactam (*n* = 2) or unknown antibiotic (*n* = 1). Statistical tests were Chi-square and ANOVA with Bonferroni post-hoc test, * *p* < 0.05

Duration of hospital stay overall was 16.4 days and significantly longer in HIV-positive patients than in those without HIV. Neurosyphilis manifestation did not influence the length of hospital stay. In six cases, complications after induction of neurosyphilis treatment was documented: candida infection (*n* = 2), thrombophlebitis (*n* = 2), exanthema (*n* = 1), IRIS (*n* = 1)). The occurrence of complications was independent of HIV status and antibiotic agent. Follow-up CSF analysis was conducted in 24 patients: only in 2 patients, CSF alterations did not improve after treatment. 22 patients showed improvement of CSF and another 23 normalization of serum parameters (RPR). For the rest, follow-up CSF or serum was not available. Reinfections were reported in seven patients who had been treated with either penicillin G (*n* = 4), ceftriaxone, doxycycline or piperacillin/tazobactam (*n* = 1 each).

## Discussion

Our analysis provides a structured and comprehensive overview of demographic, clinical, laboratory, and therapeutic characteristics in a large German cohort of patients with neurosyphilis.

Although our cohort of patients with neurosyphilis does not reflect the epidemiological increase in syphilis cases in Germany since 2010 [7] – likely due to its limited size – it mirrors several key demographic trends described in recent literature. The predominance of male patients (95%) and high proportion of HIV co-infections (43%) in our study cohort align with recent reviews, which highlight neurosyphilis as increasingly concentrated, particularly in men who have sex with men and among people living with HIV [4]. The mean age of 49 years in our cohort is also consistent with recently published European data [11]. However, comparisons across Europe reveal variability: reported proportions of male patients range from 75% [12] and 79% [11] to 89% [13], while the prevalence of HIV co-infection varies widely between 15% [12], 19% [11], and 50% [13]. Similarly, the frequency of asymptomatic neurosyphilis differs substantially across studies. Dutch data from 1999 to 2010 indicate 5% asymptomatic cases [12], and Danish data from 2015 to 2021 report 16% [11]. In contrast, the proportion in our cohort was substantially higher at 26%. A large Canadian retrospective cohort (1973–2017, *n* = 251) reported a comparable rate of asymptomatic patients at 31%, with ocular involvement and cognitive impairment being the most common manifestations – parallel to our findings [14]. The Danish cohort likewise identified early meningitis and ocular neurosyphilis as the predominant manifestations [11]. Taken together, the distribution of clinical types in our study aligns with reports from other Western countries. Nevertheless, international comparisons suggest geographic variation in clinical manifestation. A systematic review from Africa found neurosyphilis to account for 3.3% of meningitis, 3.6% of dementia and 11% of stroke cases [15]. Recent Chinese studies describe demographic patterns resembling Western cohorts – predominantly middle-aged male patients, but with markedly lower HIV co-infection rate (9%) and a different clinical profile, where general paresis predominated and ocular neurosyphilis was less common [16, 17]. As this was a retrospective single-center study, it should be added that differences in demographic and clinical characteristics compared with other cohorts may potentially reflect center-specific case mix as much as true geographic variation.

The observation that asymptomatic and ocular presentations accounted for a substantial proportion of cases is consistent with the evolving clinical spectrum of neurosyphilis in modern antibiotic-era cohorts [4]. This shift toward more subtle manifestations highlights how reliance on classic neurological symptoms alone may lead to underdiagnosis [18].

Diagnosing neurosyphilis remains challenging, as it is based on several factors such as clinical presentation, CSF changes, and positive syphilis serology in serum and CSF, without one single parameter that can reliably confirm or rule out the diagnosis. In clinical practice, treating physicians are often confronted with three diagnostically ambiguous constellations: (i) patients without CSF abnormalities, (ii) patients without evidence of intrathecal treponemal antibody synthesis or negative CSF VDRL, and (iii) patients without neurological or psychiatric symptoms.

In our study, we identified a subgroup of five patients (6.5%) who had a normal CSF cell count and protein levels, but fulfilled diagnostic criteria based on clinical presentation, serological findings, and CSF treponemal antibody synthesis. Four of them were classified as general paresis. One of them displayed a negative antibody index, suggesting residual serological activity rather than active neurosyphilis. However, he experienced clinical improvement after treatment with intravenous penicillin G. Neurosyphilis with normal CSF parameters is not uncommon: in recent Chinese cohorts, only 60% of neurosyphilis patients demonstrated abnormal CSF cell counts and protein concentrations [16, 19]. When comparing CSF leukocyte counts across neurosyphilis manifestations, values in patients with asymptomatic neurosyphilis were significantly lower than in those with symptomatic disease. Across neurosyphilis stages, CSF leukocyte counts were slightly higher in early neurosyphilis than in late types, although this difference did not reach statistical significance. These observations are consistent with evidence that neurosyphilis may occur without CSF pleocytosis or elevated CSF protein levels [4, 20], that CSF cell counts tend to be lower in asymptomatic neurosyphilis [21, 22], and that cell counts decline as the disease progresses from early to late stages [3]. Moreover, 80% of patients with normal CSF were PLWH. In advanced stages of HIV disease, less CSF pleocytosis in neurosyphilis as a blunted inflammatory response is reported [23], yet on the other hand, HIV itself can cause CSF pleocytosis [24], thereby confounding the interpretation of CSF parameters.

The second diagnostically challenging group consists of patients with probable neurosyphilis, who do not demonstrate an elevated CSF/serum treponemal antibody index. In our cohort, there were 30 patients with abnormal CSF parameters, but a negative treponemal antibody index. Among these, 15 of 22 evaluable patients showed clear clinical improvement after treatment. Moreover, in 15 patients, symptom duration ranged from 1 day to 2 months at the time of diagnosis, raising the possibility that intrathecal antibody production had not yet developed at the time of lumbar puncture.

For categorization of neurosyphilis cases, we used the diagnostic criteria of the current German guideline, as all patients were diagnosed and treated within Germany. These criteria are based on positive syphilis serology, corresponding neurological or psychiatric symptoms, abnormal CSF parameters, and clinical or serological improvement following antibiotic therapy [10]. Detection of intrathecal treponemal antibody synthesis by positive CSF/serum antibody index confirmed the diagnosis. Although the distinction between active neurosyphilis and residual serological state may be debated in some suspected or probable cases within our cohort, it is noteworthy that a substantial proportion of these patients demonstrated clinical and/or serological improvement after intravenous antibiotic therapy. These findings suggest a potential beneficial effect of antibiotic therapy even in diagnostically controversial cases.

Lastly, a quarter of our cohort was asymptomatic – without presentation of typical neurological or psychiatric symptoms. This finding aligns with other studies that report 5% to 30% asymptomatic cases [11, 12, 14]. Current CDC guidelines do not recommend routine lumbar puncture for the evaluation of latent asymptomatic neurosyphilis [25, 26]. The relatively large proportion of asymptomatic patients in our cohort likely reflects historical referral patterns as 75% of this subgroup were treated before 2015. For example, HIV-positive status or lack of titer reduction despite antibiotic treatment might have triggered neurological evaluation in these cases, as not all 2,201 patients treated for syphilis during the study period were assessed. Applying the current recommendations to our cohort, those 20 asymptomatic patients would not have been diagnosed or treated for neurosyphilis. The study design does not allow any conclusion on how many of those patients would have developed neurological symptoms without antibiotic treatment due to missing long-term follow-up. This scenario can be debated either as misdiagnosis or over-treatment. The first concern is further supported by a large systematic review including more than 7,000 patients from China, in which over half of neurosyphilis cases were initially misdiagnosed [16]. Along this line, Arns et al. recently identified VDRL titers > 1:32 as a risk factor for neurosyphilis – in addition to the presence of neurological symptoms and HIV viral load > 400 copies/µl [27]. Considering historical data indicating that 4% to 9% of untreated syphilis cases progress to symptomatic neurosyphilis [28] and that approximately 35% of asymptomatic neurosyphilis patients may develop symptomatic disease over time [21], timely diagnosis and effective treatment are essential. On the other hand, the clinical significance of CSF laboratory abnormalities in patients without any neurologic findings is uncertain [29].

An important subgroup of neurosyphilis patients are HIV-positive individuals. Our observation that CSF abnormalities (pleocytosis, elevated protein) and co-infections were more pronounced in HIV-positive patients aligns with previous evidence indicating that HIV enhances CNS inflammatory responses and susceptibility to opportunistic pathogens [30, 31]. Notably, ocular neurosyphilis was less frequent among HIV-positive individuals in our cohort. A predomination of specific neurosyphilis manifestations in people with HIV has not been consistently demonstrated in the literature. A large Canadian cohort of 251 patients described a higher likelihood of early neurosyphilis among those with HIV co-infection [14]. In contrast, recent Chinese studies identified meningovascular and asymptomatic presentations as the predominant types in HIV-positive patients [22, 32], whereas an American cohort reported asymptomatic and ocular neurosyphilis as the most common manifestations in this population [33].

Furthermore, we observed that nearly half of the confirmed neurosyphilis cases had a negative CSF VDRL result — a limitation previously described in neurosyphilis diagnostics with a sensitivity of only approximately 50% [3, 10]. Consequently, CSF VDRL cannot be relied upon as a standalone diagnostic tool, underscoring the need for more sensitive and specific new biomarkers. Several candidates have emerged in recent research, including chemokines such as CXCL13, CXCL10, and CXCL8 [4, 34] – although CXCL-13 is also an important biomarker for neuroborreliosis [35]. Additional potential biomarkers under investigation include serum homocysteine [36] and immune-based markers such as the neutrophil CD64 index and neutrophil to lymphocyte ratio (NLR) [37].

Therapeutically, intravenous application of penicillin G is recommended as first line therapy for neurosyphilis by international and German guidelines. Notably, while the German guideline recommends ceftriaxone as main alternative, the CDC recommends procaine penicillin G plus probenecid and restricts ceftriaxone to situations involving penicillin allergy [10, 29]. A recent French multicenter study compared ceftriaxone to benzylpenicillin treatment and reported equivalent efficacy with shorter hospital stay among ceftriaxone-treated patients, supporting its use as a practical alternative in clinical settings [38]. Therefore, the predominance of penicillin G with ceftriaxone serving as main alternative in our cohort aligns with guideline-based practice. Because ceftriaxone was never administered to asymptomatic patients, we hypothesized that it was primarily initiated empirically in cases of suspected CNS infections of unclear etiology – in line with the guideline recommendations on acute bacterial meningitis [39] – and subsequently continued once neurosyphilis was diagnosed. Doxycycline was used in three patients in our cohort, mostly due to penicillin allergy. Although doxycycline has shown promising results in several studies [40, 41], it is not yet formally recommended by current clinical guidelines due to absence of randomized controlled trials and robust longitudinal data on long-term outcomes. However, the German guideline explicitly states that doxycycline (2 × 200 mg per day for 28 days) may be considered as an alternative regimen in selected cases [10]. Guidelines currently recommend a treatment duration of 10–14 days for intravenous penicillin G. Given the occurrence of relapses or serofast states, Luo et al. compared additional weekly benzathine penicillin injections after standard therapy 14-day i.v. penicillin G for either 3 weeks or > 4 weeks and found that cure – defined as > 4-fold RPR decline or reversion to nonreactive – in their cohort of 264 HIV-negative patients was more frequent in the prolonged group at 6 and 12 months [42]. Thus, further studies on improving cure rates by extending the duration of antibiotic treatment would be of interest.

As a single-center, retrospective study, our findings may be subject to referral bias and incomplete data capture. For example, patients with ocular neurosyphilis in our cohort were partly diagnosed in the department of ophthalmology, therefore we cannot completely rule out misdiagnosed patients with ocular neurosyphilis who may not be represented in this cohort. Due to the age of part of the documentation, comprehensive information on transsexuality is lacking. Subgroup analyses (e.g., by sex or specific neurosyphilis subtype) are underpowered due to the relatively small and heterogenous sample size of the cohort. Some CSF or imaging data were missing, and long-term follow-up was limited. Future prospective multicenter cohorts, possibly incorporating novel biomarkers (e.g., CXCL13), are needed to validate diagnostic thresholds and correlate laboratory findings with long-term neurologic outcomes.

In summary, within this large German cohort, neurosyphilis most frequently presented as asymptomatic or ocular disease, while late-stage manifestations such as general paresis and tabes dorsalis were relatively uncommon. Diagnostic evaluation proved challenging due to the broad clinical spectrum, the occasional absence of classical CSF abnormalities, and the limited sensitivity of CSF VDRL, emphasizing the need for multimodal diagnostic approaches and an international diagnosis standard. A noteworthy proportion of asymptomatic cases would not have been detected under current guideline recommendations, underscoring the complexity of identifying early or subtle types of neurosyphilis in clinical practice. Despite diagnostic uncertainty in some patients, many demonstrated clinical or serological improvement following antibiotic therapy, highlighting the importance of timely recognition and treatment. Lastly, as the incidence of syphilis continues to rise, maintaining clinical vigilance and optimizing diagnostic pathways will be crucial to preventing irreversible neurological sequelae.

## Supplementary Information

Below is the link to the electronic supplementary material.

Supplementary Material 1.

## Data Availability

The data that support the findings of this study are available from the corresponding author upon reasonable request.
