# Supplementary material for: Clinical presentation of neurosyphilis – a single-center retrospective data analysis
Source: Neurol Res Pract. 2026 Apr 30;8(1):35. doi: 10.1186/s42466-026-00497-1 (PMC13134295; doi:10.1186/s42466-026-00497-1)
Supplement: Supplementary file 1 — Supplementary Material 1. [file 42466_2026_497_MOESM1_ESM.docx]

**Supplemental material**

| **Data** | **1 : early meningitis** | **2 :meningo-vascular neuro-syphilis** | **3 : ocular neuro-syphilis** | **4 : tabes dorsalis** | **5 : general paresis** | **6 :asymp-tomatic neuro-syphilis** |
| --- | --- | --- | --- | --- | --- | --- |
| **Age**, years, median (IQR) | 46 (39-61) | 47 (37-51) | 44 (39-49) | 54 (44-65) | **61 (54-66)*** vs. 2/3/6 | 47 (40-53) |
|  |  |  |  |  |  |  |
| **CSF parameters** |  |  |  |  |  |  |
| Cells/µl, median (IQR) | 15 (8-43) | 16 (6-86) | 35 (7-76) | 8 (1-8) | 4 (4-22) | 9 (6-14) |
| Lymphocytes (%),median (IQR) | 73 (62-87) | 86 (82-92) | 81 (66-89) | 93 (75-95) | 80 (69-85) | 78 (62-85) |
| Total protein (mg/dl), median (IQR) | 70.2 (51.8-89.6) | 68.2 (33.2-142) | 52.5 (38.9-69.5) | 48.9 (36.3-67.3) | 70.0 (40.0-220) | 44.3 (35.0-62.0) |
| Albumin index, median (IQR) | 9.4 (8.2-14.3) | 11.4 (7.2-23.5) | 7.8 (5.6-11.4) | 7.5 (5.2-9.9) | 10.4 (5.6-21.9) | 6.9 (4.6-8.5) |
| OCB pos. | 4/6 (66.7%) | 8/10 (80.0%) | 12/18 (66.7%) | 4/5 (80.0%) | 6/9 (66.7%) | 11/16 (68.8%) |
| Glucose (mg/dl), median (IQR) | 59.9 (50.9-70.9) | 51.3 (42.3-58.3) | 62.9 (56.1-69.7) | 62.4 (59.0-70.5) | 61.0 (46.5-68.0) | 62.9 (59.9-71.8) |
| Glucose-index, median (IQR) | 0.61 (0.43-0.76) | 0.47 (0.36-0.67) | 0.56 (0.51-0.63) | 0.56 (0.50-0.62) | 0.59 (0.42-0.67) | 0.59 (0.54-0.72) |
| Lues IgG index, median (IQR) | 1.2 (0.8-4.4) | 7.6 (1.2-16.1) | 2.5 (1.0-8.8) | 1.2 (0.7-5.2) | 11.7 (1.8-31.6) | 2.0 (0.9-8.7) |
|  |  |  |  |  |  |  |
| **Serum parameters** |  |  |  |  |  |  |
| CRP (mg/dl), median (IQR) | 0.2 (0.1-0.3) | 1.3 (0.3-2.5) | 0.5 (0.1-1.9) | 0.4 (0.2-1.7) | 0.3 (0.1-4.6) | 0.3 (0.2-1.9) |
| Leukocytes (G/l), median (IQR) | 6.4 (5.5-6.7) | 6.3 (4.2-7.6) | 7.3 (5.6-10.1) | 6.6 (4.9-9.4) | 6.1 (4.8-9.0) | 6.13 (4.5-7.4) |
| TPPA pos. | 7 | 12 | 20 | 6 | 12 | 20 |
| FTA-Abs pos. | 5 | 8 | 14 | 4 | 9 | 15 |
| Cardiolipin-RPR pos. | 7/7 (100.0%) | 10/12 (83.3%) | **20/20 (100.0%)*** | **3/6 (50.0%)*** | 8/12 (66.7%) | 16/17 (94.1%) |
|  |  |  |  |  |  |  |
| **Additional pathogens** | 1 | 3 | 0 | 1 | 2 | 0 |
|  |  |  |  |  |  |  |
| **cMRI abnor-malities**, n (%) | 0/3 (0.0%) | 3/5 (60.0%) | **1/11 (9.1%)*** | 3/4 (75.0%) | **7/8 (87.5%)*** | 0/2 (0.0%) |
|  |  |  |  |  |  |  |
| **Diagnostic criteria fulfilled** |  |  |  |  |  |  |
| Probable neurosyphilis | 5 | 4 | 9 | 3 | 3 | 10 |
| Confirmed neurosyphilis | 2 | 8 | 11 | 3 | 9 | 10 |

**Supplemental table 1: Diagnostic findings depending on neurosyphilis form.** Statistical tests were Chi-square test and ANOVA with Bonferroni post-hoc test, * p<0.05.

| **Treatment and outcome** | **HIV-positive (n=33)** | **HIV-negative (n=44)** | **All patients (n=77)** |
| --- | --- | --- | --- |
| Penicillin G | 24 (72.7%) | 31 (70.5%) | 54 (70.1%) |
| Ceftriaxone | 7 (21.2%) | 9 (20.5%) | 16 (20.8%) |
| Doxycycline | 1 (3.0%) | 2 (4.5%) | 3 (3.9%) |
| Other antibiotic | 1 (3.0%) | 2 (4.5%) | 4 (5.2%) |
|  |  |  |  |
| Duration of hospital stay (days), mean±std | **18.1±7.0*** | **15.0±6.3*** | 16.4±6.8 |
| Complications, n (%) | 2/33 (6.0%) | 5/44 (11.4%) | 7/77 (9.1%) |
| Clinical improvement, n (%) | 21/24 (87.5%) | 21/24 (87.5%) | 42/48 (87.5%) |

**Supplemental table 2: Treatment and outcome according to HIV status.** Statistical tests were student’s t-test, Mann-Whitney test and Chi-square test, * p<0.05.
